# Tilapia Lake Virus-Induced Neuroinflammation in Zebrafish: Microglia Activation and Sickness Behavior

**DOI:** 10.3389/fimmu.2021.760882

**Published:** 2021-10-11

**Authors:** Miriam Mojzesz, Magdalena Widziolek, Mikolaj Adamek, Urszula Orzechowska, Piotr Podlasz, Tomasz K. Prajsnar, Niedharsan Pooranachandran, Anna Pecio, Anna Michalik, Win Surachetpong, Magdalena Chadzinska, Krzysztof Rakus

**Affiliations:** ^1^ Department of Evolutionary Immunology, Institute of Zoology and Biomedical Research, Faculty of Biology, Jagiellonian University, Krakow, Poland; ^2^ Fish Disease Research Unit, Institute for Parasitology, University of Veterinary Medicine, Hannover, Germany; ^3^ Department of Pathophysiology, Forensic Veterinary Medicine and Administration, Faculty of Veterinary Medicine, University of Warmia and Mazury, Olsztyn, Poland; ^4^ Department of Comparative Anatomy, Institute of Zoology and Biomedical Research, Faculty of Biology, Jagiellonian University, Krakow, Poland; ^5^ Department of Invertebrate Development and Morphology, Institute of Zoology and Biomedical Research, Faculty of Biology, Jagiellonian University, Krakow, Poland; ^6^ Department of Veterinary Microbiology and Immunology, Faculty of Veterinary Medicine, Kasetsart University, Bangkok, Thailand

**Keywords:** anti-viral response, interferons, neuroinflammation, microglia, sickness behavior, tilapia lake virus, TiLV, zebrafish

## Abstract

In mammals, the relationship between the immune system and behavior is widely studied. In fish, however, the knowledge concerning the brain immune response and behavioral changes during brain viral infection is very limited. To further investigate this subject, we used the model of tilapia lake virus (TiLV) infection of zebrafish (*Danio rerio*), which was previously developed in our laboratory. We demonstrated that TiLV persists in the brain of adult zebrafish for at least 90 days, even when the virus is not detectable in other peripheral organs. The virions were found in the whole brain. During TiLV infection, zebrafish displayed a clear sickness behavior: decreased locomotor activity, reduced food intake, and primarily localizes near the bottom zone of aquaria. Moreover, during swimming, individual fish exhibited also unusual spiral movement patterns. Gene expression study revealed that TiLV induces in the brain of adult fish strong antiviral and inflammatory response and upregulates expression of genes encoding microglia/macrophage markers. Finally, using zebrafish larvae, we showed that TiLV infection induces histopathological abnormalities in the brain and causes activation of the microglia which is manifested by changes in cell shape from a resting ramified state in mock-infected to a highly ameboid active state in TiLV-infected larvae. This is the first study presenting a comprehensive analysis of the brain immune response associated with microglia activation and subsequent sickness behavior during systemic viral infection in zebrafish.

## 1 Introduction

During infection or injury, the host defense mechanisms are activated, and this induces not only immune response and profound physiological changes in the organism (for example, induction of fever) but also behavioral changes, the so-called sickness behavior. These behavioral changes include lethargy, hyperalgesia, sleepiness, reduced food intake, depression of locomotor, exploratory, and social activity, cognitive and memory deficits, disorientation, and reduced sexual activity (1–4). Sickness behavior evolved not only to save the energy necessary to fight the infection and to facilitate the healing process but also to minimize the risk of infection spread or predation (5). Sickness behavior is triggered by proinflammatory mediators, mainly cytokines (IL-1β, IL-6, TNF-α, and type I interferons (IFN)), and prostaglandins (e.g., PGE2), which are secreted in response to acute infections and/or tissue injury, and might pass the blood-brain barrier, activate glial cells (astrocytes and microglia), and promote behavioral changes (1, 6).

Microglia and astrocytes are responsible for the local innate immune response in the brain. These cells are rapidly activated in response to brain inflammation, infection, and injury (7–9). Even upon a minor infection, microglia change the gene expression profile. In addition, the morphology of microglia also changes from a ramified, resting phenotype dynamically scanning the brain into amoeboid motile cells which subsequently migrate to the affected site (10). In mammals, activation of microglia during viral infection is well documented (11). In fish, however, little is known about the microglia activation during viral infection of brain, although neurotropic viruses might potentially affect its activity/function.

Zebrafish (*Danio rerio*) is a laboratory model organism used for translational studies of human and animal diseases, including viral infections (12–14). However, the knowledge regarding the brain immune response and behavioral changes during brain viral infection of zebrafish is very limited. Only few reports described the immune response of zebrafish brain during infection with nervous necrosis virus (NNV) and spring viremia of carp virus (SVCV) (15, 16). Moreover, it was also shown that SVCV infection or poly(I:C) stimulation induces behavioral fever in zebrafish (17, 18). Besides, zebrafish has also been used to study the brain entry routes of human viruses infecting the central nervous system (CNS), such as Sindbis virus (SINV), Chikungunya virus (CHIKV), and Herpes simplex virus type 1 (HSV-1) (19–21).

In recent years, tilapia lake virus (TiLV; genus: *Tilapinevirus*, family *Amnoonviridae*) was described as an etiological agent of highly contagious and emerging disease that has detrimental effects on tilapia farming worldwide (22–24). TiLV has a linear, negative-sense single-strand RNA genome with 10 segments and about 10.323 kb in total length (22, 23). Interestingly, a high level of TiLV was observed in the brain of infected tilapia (23, 25), and this was correlated with the clear histopathological changes (23) and activation of the expression of genes involved in the antiviral immune response in the tilapia brain (25, 26). We recently described the TiLV infection model in adult and larvae of zebrafish (27, 28), and our virus tropism study revealed that in adult fish the highest viral load of TiLV was found in the brain (27).

In the present study, we demonstrate that TiLV persists in the brain of adult zebrafish for a long time and that during TiLV infection, zebrafish expresses clear sickness behavior: decreased locomotor activity and food intake. Moreover, TiLV induces in the brain histological changes, strong antiviral and inflammatory response, and activates microglia/macrophages.

## 2 Materials and Methods

### 2.1 Virus and Cells

TiLV (VETKU-TV01 isolate) was previously isolated from red hybrid tilapia (*Oreochromis* spp.) in Thailand (29). The E-11 cells from the striped snakehead fish (*Ophicephalus striatus*) were used to produce and titrate TiLV as described earlier (27).

### 2.2 Zebrafish Maintenance

Zebrafish (*Danio rerio*) were grown in 8 L tanks on a ZebTEC Stand Alone system (Tecniplast, Buguggiate, Italy) at a water temperature of 28°C and a day/night cycle of 12/12 h. Before the infection, adult zebrafish were placed into 30 L single aquaria with aeration and water temperature of 28°C for 2 weeks of acclimatization. Fish were fed twice per day with commercial feed for zebrafish (Gemma Micro 300ZF, Skretting, Stavangar, Norway). Zebrafish larvae were obtained by incross and maintained in E3 medium with methylene blue at 28°C according to standard protocols. The animal study was accredited by the 2nd Local Ethics Committee in Kraków, Poland (No. 29/2021).

### 2.3 TiLV Infection and Sample Collection

#### 2.3.1 Adult Zebrafish

Prior to infection, adult zebrafish (Tuebingen strain, TU) were anesthetized in water containing 0.2 g/L of buffered solution of MS-222 (Sigma-Aldrich, ST. Louis, MO, USA) and injected intraperitoneally (i.p.) with 10 μl of medium containing TiLV (1 × 10^7^ TCID50/ml). Mock-infected controls were treated similarly and injected i.p. with L15 medium collected from noninfected cells. At selected time points: day 0 and 1, 3, 6, 14, 28, 45, 60, and 90 days postinfection (dpi), infected fish (*n* = 7–8) were euthanized and sampled for the viral load and gene expression study. Organs (brain, eye, spleen, kidney, liver) were collected into fixRNA (EURx) and stored at −20°C until RNA isolation. Moreover, at 14 dpi, additional infected fish (*n* = 4) were sampled to collect three parts of the brain (forebrain, midbrain, and hindbrain) for viral load and electron microscopy study.

#### 2.3.2 Zebrafish Larvae

Prior to infection, zebrafish larvae at 54 h postfertilization (hpf): Tuebingen strain for viral load and gene expression study, and transgenic line *Tg(mpeg1.1:mCherryF)ump2* (30) for microscopic evaluation of microglia activation, were anesthetized and microinjected systemically with 3 nl of medium containing TiLV (1 × 10^7^ TCID50/ml) or control L15 medium (mock-infection) into the duct of Cuvier as described previously (28). At 24 and 48 hpi, larvae were anesthetized and used for (i) sample collection: heads and the rest of the bodies were collected separately into the fixRNA (EURx) (eight larvae pooled per sample) and stored at −20°C until RNA isolation and (ii) microscopic evaluation of microglia activation.

### 2.4 RNA Isolation and cDNA Synthesis

Total RNA was isolated by using the ReliaPrep™ RNA Tissue Miniprep System (Promega, Madison, WI, USA) according to the manufacturer’s instruction. The purity and concentration of RNA were measured spectrophotometrically with a Tecan Spark reader using a NanoQuant plate (Tecan, Männedorf, Switzerland). Synthesis of cDNA was performed from 100 ng of total RNA using the High-Capacity cDNA Reverse Transcription Kit (Applied Biosystems, Waltham, MA, USA) according to the manufacturer’s instructions. For selected samples, no-reverse transcriptase (no-RT) controls were used to check for genomic DNA contamination.

### 2.5 Real-Time qPCR

#### 2.5.1 Viral Load Analysis

The approximation of viral load was performed by quantification of normalized gene copies as described earlier (27). The approximation of viral load is shown as the copy number of the TiLV gene normalized against 1 × 10^5^ copies of the host reference gene, elongation factor 1 alpha (*ef1α*). The genes analyzed and the sequences of their respective primers are presented in **Table 1**.

**Table 1:** Primers and probes used for RT-qPCR.

Gene		Primer sequence (5’→3’)	Reference or accession number
*TiLV*	F	AGCCTGCCACACAGAAG	(27)
	R	CTGCTTGAGTTGTGCTTCT	
	P	FAM-CTCTACCAGCTAGTGCCCCA-BHQ	
*ef1a*	F	TGCCAGTGTTGCCTTCGT	(27)
	R	GCTCAATCTTCCATCCCTTG	
*rps11*	F	TAAGAAATGCCCCTTCACTG	NM_213377.1
	R	GTCTCTTCTCAAAACGGTTG	
*rig-I*	F	TTGAGGAGCTGCATGAACAC	JX462558.1
	R	CCGCTTGAATCTCCTCAGAC	
*tlr3*	F	AAAGGGCTACGTTTGGTGTG	NM_001013269.3
	R	GTTGGTGGAGTTCAGCCATT	
*irf3*	F	CAAAACCGCTGTTCGTGCC	(31)
	R	CATCGTCGCTGTTGGAGTCCT	
*irf7*	F	AGGCAGTTCAACGTCAGCTACCAT	(31)
	R	TTCCACCAAGTTGAGCAATTCCAG	
*infφ1*	F	GAGCACATGAACTCGGTGAA	NM_207640.1
	R	TGCGTATCTTGCCACACATT	
*mxa*	F	GACCGTCTCTGATGTGGTTA	AJ544823.1
	R	GCATGCTTTAGACTCTGGCT	
*il-1β*	F	GAACAGAATGAAGCACATCAAACC	NM_212844.2
	R	ACGGCACTGAATCCACCAC	
*ifnγ1-2*	F	CTATGGGCGATCAAGGAAAA	AB158361.1
	R	CTTTAGCCTGCCGTCTCTTG	
*tnf-α (cxcl8a)*	F	GCGCTTTTCTGAATCCTACG	NM_212859.2
	R	TGCCCAGTCTGTCTCCTTCT	
*il-8*	F	GTCGCTGCATTGAAACAGAA	XM_009306855.3
	R	CTTAACCCATGGAGCAGAGG	
*cox2b*	F	CCCCAGAGTACTGGAAACCA	NM_001025504.2
	R	ACATGGCCCGTTGACATTAT	
*il-10*	F	ATTTGTGGAGGGCTTTCCTT	NM_001020785.2
	R	AGAGCTGTTGGCAGAATGGT	
*gfap*	F	AGTCCCAGCGTTCCTTCTC	NM_131373.2
	R	GGTCGTTTAGCCCCATCA	
*csf1r*	F	CCTGATCCGCAACGTTCATCCT	NM_131672.1
	R	GCTTTGGGCAGCATTCTTGAGG	
*apoeb*	F	AGATGGGAGGAGATGGTGGA	NM_131098.2
	R	GGAGCCCTTGATGTTTTGC	
*cd68*	F	CACCACACAGGCTACTGACG	XM_002662803.5
	R	AATCCGTGCTTCATTTTCGT	
*npy*	F	CTGTGATGTCCATGTGTCCTTCTG	NM_131074.2
	R	GAGCCTAAAGAGCGCACATTGA	
*pomc*	F	CAGAGTCTGAGCTTGGGTTTGCTT	NM_001083051.1
	R	ACTTTTACCGGTCTGCGTTTGC	
*tmem119a*	F	GCGAGAACCTACGAAAGCAC	XM_682863.6
	R	CACCACTTCAACATCCTCCA	
*tmem119b*	F	CATCTTCACCATCCTCTGCTC	NM_001089443.1
	R	GCCTTGTCTTGCTCATCCAC	
*aif1*	F	TGAAGACAAAAAGGGAGAAGC	NM_198870.1
	R	GCACGCACACACAAACATAA	

#### 2.5.2 Gene Expression Analysis

For gene expression analysis, the real-time qPCR (RT-qPCR) reactions were performed in duplicate for each sample using SYBR Select Master Mix (Applied Biosystems), specific primers, and cDNA (from larvae: 20 × diluted; from adult fish organs: 40 × diluted). Reaction mix and amplification protocol were as described previously (27). The melting curve analyses were performed at the end of each run. No-RT and nontemplate controls were included in selected runs. RT-qPCR was carried out with a Rotor-Gene Q (Qiagen, Hilden, Germany). Infection-induced changes in the gene expression were rendered as a ratio of target gene *vs.* reference gene (*rps11*; ribosomal protein s11 gene) relative to expression in control samples using the Pfaffl method (32) according to the following equation:


$$Ratio=\frac{{\left(E_{target}\right)}^{\Delta Ct}Target^{\left(control-sample\right)}}{{\left(E_{reference}\right)}^{\Delta Ct}Reference^{\left(control-sample\right)}}$$

where *E* is the amplification efficiency and Ct is the number of PCR cycles needed for the signal to exceed a predetermined threshold value. The genes analyzed and the sequences of their respective primers are presented in **Table 1**.

### 2.6 Behavioral Study

#### 2.6.1 Behavioral Evaluation

To assess the behavioral changes caused by TiLV in adult zebrafish, three tanks (3.5 L, 11 cm × 17 cm × 28 cm; width × depth × length) with TiLV-infected fish (five fish in each tank) and three tanks with mock-infected fish (five fish in each tank) were used for recording the videos by a camera (NIKON Coolpix L340) located in front of the tank. After setting the tank in the recording place, fish were left undisturbed for 5 min. Next, the fish behavior was recorded in each tank for 29 min at 6 and 11 dpi. Mock-infected and TiLV-infected tanks were recorded in alternating order. The videos were analyzed using software EthoVision XT 15 (Noldus, Wageningen, Netherlands). The following parameters were analyzed: velocity (the distance moved by the center, nose or tail-base point of the subject per unit time when fish were in-motion) (cm/s), distance traveled (the distance traveled by the center, nose or tail-base point of the subject from the previous sample to the current one)(cm), time in motion/motionless (s), and percentage of time spent in the top zone *vs.* bottom zone of the tank (%).

#### 2.6.2 Zebrafish Weight Evaluation

During the course of the experiments, fish were fed twice per day with commercial feed for zebrafish (Gemma Micro 300ZF, Skretting). The weight of mock-infected (*n* = 10) and TiLV-infected (*n* = 10) zebrafish was evaluated every 3–4 days starting from 14 days before infection until 25 dpi. Fish were weighed individually with precision balance (WTB 2000, Radwag).

### 2.7 Histopathology

Six specimens (3 mock-infected and 3 TiLV-infected) of zebrafish larvae at 48 hpi (102 hpf) were euthanized with an overdose of 1% MS-222 and fixed in 10% PBS-buffered formalin. Then, the larvae were rinsed in tap water for 4 hours, dehydrated in graded alcohol, infiltrated in a mixture 1:1 propylene oxide/Epon 812 and embedded in Epon 812. The whole heads were sectioned transversely at 0.5-1 µm thickness using glass knives. Sections were stained with methylene blue/azur II and analyzed under Nikon Eclipse E600. Selected sections of the forebrain, midbrain, and hindbrain were digitalized using the NIS-Elements F software and post-processed by CorelDraw Graphic Suite 2020.

### 2.8 Transmission Electron Microscopy Analysis

Dissected brains were fixed in 2.5% glutaraldehyde solution in 0.1 M phosphate buffer (pH 7.4) at 4°C for 2 weeks. After this time, samples were washed with 0.1 M phosphate buffer with the addition of sucrose (5.8%), postfixed in 1% solution of osmium tetroxide, dehydrated in ethanol and acetone series, and embedded in epoxy resin Epon 812 (SERVA, Heidelberg, Germany). For ultrastructural analyses, the resin blocks were cut into ultrathin sections, contrasted with lead citrate and uranyl acetate, and observed under the JEOL JEM 2100 transmission electron microscope.

### 2.9 Microscopic Analysis of Microglia Activation

To analyze microglia activation upon TiLV infection, the transgenic *Tg(mpeg1.1:mCherryF)ump2* zebrafish larvae infected with TiLV or mock-infected, were anesthetized at 24 and 48 hpi, and positioned dorsally in 1% low-melting point agarose (Sigma-Aldrich) in E3 w/o methylene blue on a glass bottom dish filled with E3 medium containing 0.1 mg/ml of MS-222 (Sigma-Aldrich). Midbrain region of each larvae was imaged with the Zeiss LSM 900 Airyscan 2 confocal microscope using the C-Apochromat 40x/NA 1.2 water objective. For image analysis, ImageJ/Fiji was used. Maximum intensity projections were created, and fluorescence photomicrograph were converted to representative binary images using previously described protocol to quantify microglia morphology (33). Then, the circularity index (0–1) was analyzed as a 4-pi (area/perimeter^2) formula, where the value of 1.0 indicates a perfect circle. Each image was additionally manually verified for the correct microglia outliers and any artifacts rejected from the analysis.

### 2.10 Statistical Analysis

Statistical analysis was performed using GraphPad Prism 9 (GraphPad Software). Shapiro-Wilk test (for normality) and Brown-Forsythe test (for homogeneity) were performed to ensure the suitability of the data for parametric significance tests. Significant differences in the gene expression between the control adult fish at day 0 and TiLV-infected adult fish in following sampling points were assessed using one-way ANOVA followed by Dunnett’s multiple comparisons test in cases when the data were normally distributed, or with the non-parametric Kruskal–Wallis test followed by Dunn’s multiple comparison test when the data were not normally distributed. Significant differences in the gene expression between the mock-infected and TiLV-infected larvae at each time point were assessed by two-way ANOVA followed by Bonferroni test. Significant differences in the velocity, distance traveled, time in-motion/motionless, time spent in the tank zones, and weight between the mock-infected and TiLV-infected adult fish in each time point were assessed by two-tailed Mann-Whitney *U* test. Significant differences in the circulatory index between the mock-infected and TiLV-infected larvae in each time point were assessed by two-tailed Mann-Whitney test. Data are presented as means (+ standard deviation). The significance levels are indicated with asterisks: ^*^ *p* ≤ 0.05; ^**^ *p* ≤ 0.01; ^***^ *p* ≤ 0.001; ^****^ *p* ≤ 0.0001.

## 3 Results

### 3.1 Brain Is the Organ With the Highest Viral Load and With the Longest Persistence of TiLV in Adult Zebrafish

**Figure 1:**
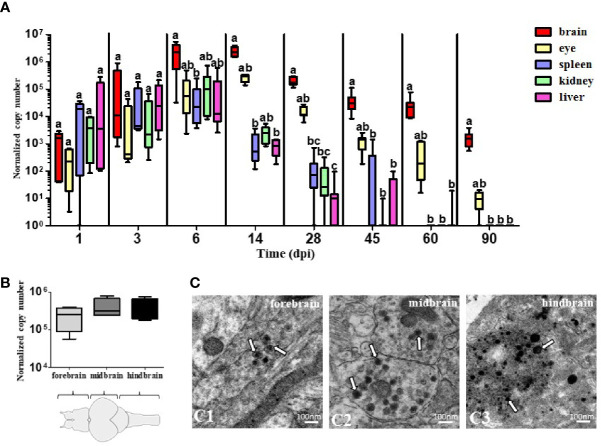
The presence of TiLV in adult zebrafish tissues including brain. **(A, B)** Normalized copy numbers of TiLV RNA in different tissues (*n* = 5–7) **(A)** and in three parts of the brain at 14 dpi (*n* = 4) **(B)** of i.p. infected zebrafish. The data are shown as box and whisker plots indicating the range of 25%–75% in the box and maximum and minimum values by whiskers. The line in the middle of the box is plotted at the median. The different letters indicate significant differences between tissues at particular time points revealed by one-way ANOVA followed by Tukey’s multiple comparison test or by the nonparametric Kruskal-Wallis test followed by Dunn’s test. *p*-value ≤ 0.05 was considered significant. Zebrafish brain scheme was obtained from scidraw.io (doi.org/10.5281/zenodo.3926217) **(C)**. Transmission electron micrographs of the brain from TiLV-infected adult zebrafish showing cytoplasmic viral particles (white arrows) in forebrain **(C1)**, midbrain **(C2)**, and hindbrain **(C3)**.

Quantification of TiLV by RT-qPCR in the brain, eye, spleen, kidney, and liver of i.p. infected adult zebrafish showed that the viral load in the organs increased progressively until 6 dpi (for the spleen, kidney, and liver) and 14 dpi (for the brain and eye), before gradually decreasing at subsequent time points (**Figure 1A**). The highest viral load was observed in the brain at 14 dpi (the mean of the normalized copy number was 2.47 × 10^6^). Interestingly, the viral load in the brain was still at a high level even at 90 dpi, when the virus was not detected in peripheral organs such as the spleen, kidney, and liver. The viral load in the eyes was at a moderate level and was also detected at 90 dpi. There were no statistically significant differences in viral load measured at 14 dpi in the three regions of the brain: forebrain, midbrain, and hindbrain (**Figure 1B**). We also confirmed the presence of TiLV particles in the three parts of the brain by transmission electron microscopy (TEM) (**Figure 1C**). The size of the viral particles was of approximately 70–100 nm. Collectively, these results suggest that TiLV is able to invade the brain of adult zebrafish and remain in the CNS for a substantial period of time.

### 3.2 TiLV Activates Expression of the Immune-Related Genes in the Brain of Adult Zebrafish and Induces Brain Inflammation

**Figure 2:**
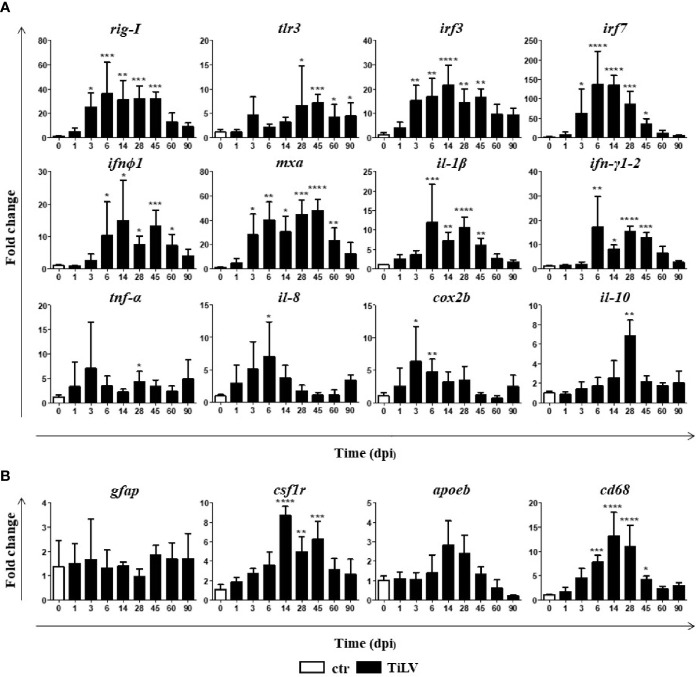
TiLV-induced changes in the expression of genes involved in antiviral response and microglia/macrophage markers in the brain of adult zebrafish. **(A, B)** Expression of genes involved in the antiviral and proinflammatory response **(A)**, microglia/macrophages, and astrocytes markers **(B)** in the brain of zebrafish. The gene expression is normalized against the housekeeping gene *rps11*. Changes in gene expression of TiLV-infected fish (black bars) are shown as *x*-fold increase compared with control fish at day 0 (white bars). The symbol (^*^) indicates significant differences between the control and TiLV-infected fish as revealed by one-way ANOVA followed by Dunnett’s multiple comparison test or by the nonparametric Kruskal-Wallis test followed by Dunn’s test (^*^ *p* ≤ 0.05; ^**^ *p* ≤ 0.01; ^***^ *p* ≤ 0.001; ^****^ *p* < 0.0001). The data are the mean + SD of *n* = 5–7 fish.

Having determined the lasting TiLV presence in the brain of adult zebrafish, we speculated whether it leads to the immune response. We found that TiLV infection induced an upregulation of the expression of genes encoding proteins involved in type I IFN pathway such as the pattern recognition receptors (PRR): *rig-I* and *tlr3*, transcription factors: *irf3* and *irf7*, type I IFN (*ifnϕ1*), and the antiviral protein Mxa (*mxa*). Upregulation of the expression of most of these genes was clearly associated with the viral load in the brain and was observed from 3 or 6 dpi till 45 or 60 dpi (**Figure 2A**). Moreover, TiLV infection induced significant upregulation of the genes encoding proinflammatory cytokines *il-1β*, *ifnγ1-2* (from 6 till 45 dpi), *tnf-α* (at 28 dpi), *il-8* (*cxcl8a*) (at 6 dpi), enzyme *cox2b* (at 3 and 6 dpi), and anti-inflammatory cytokine *il-10* (at 28 dpi) (**Figure 2A**). Finally, we studied the expression of the activation markers of astrocytes: glial fibrillary acidic protein (*gfap*) and microglia/macrophages: colony-stimulating factor 1 receptor (*csf1r*), apolipoprotein EB (*apoeb*), and *cd68*. Upregulation of the expression of *csf1r* (from 14 till 45 dpi) and *cd68* (from 6 till 45 dpi) was observed. The expression of *apoeb* appeared increased, although not statistically significant, whereas no differences in the expression of *gfap* were observed (**Figure 2B**). We also studied expression of other microglia/macrophage markers such as allograft inflammatory factor 1 (*aif1*, also known as ionized calcium-binding adapter molecule 1 (IBA1)), which is a well-known marker of microglia in mice and transmembrane protein 119 (*tmem119*), but the expression of these genes was not changed (data not shown). Together, these data indicate that TiLV induces antiviral response in the brain of adult zebrafish and upregulation of some of the microglia/macrophage markers.

### 3.3 TiLV Induces Sickness Behavior and Leads to Weight Loss in Adult Zebrafish

**Figure 3:**
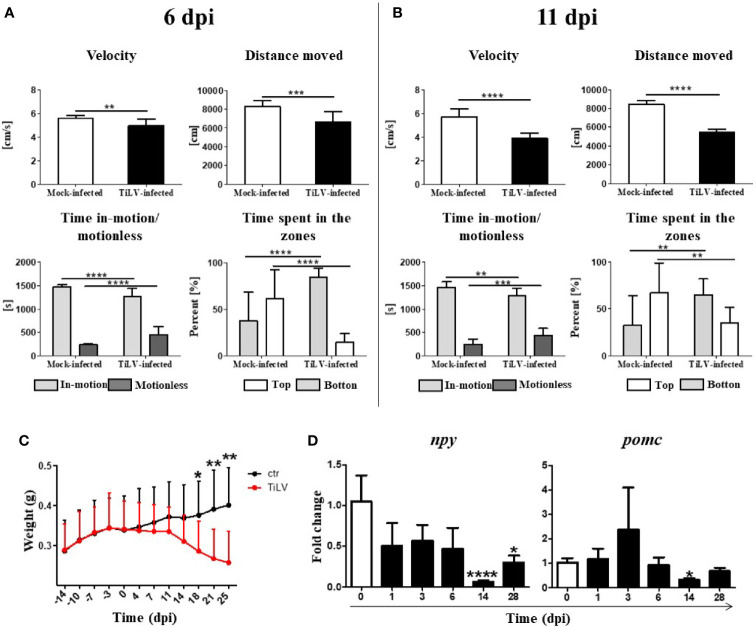
TiLV-induced changes in locomotor activities and feed intake in adult zebrafish. Behavior parameters tests of mock-infected and TiLV-infected fish at 6 dpi **(A)** and 11 dpi **(B)**. Results in **(A, B)** are representative of two independent experiments. Body weight (g) of mock-infected and TiLV-infected adult zebrafish **(C)**. The symbol (^*^) indicates significant differences between the mock-infected and TiLV-infected fish as revealed by two-tailed nonparametric Mann-Whitney test. The data are the mean + SD of *n* = 15 (behavioral parameters tests) or *n* = 9–11 (body weight test). **(D)** Expression of *npy* and *pomc* in the brain of adult zebrafish. The gene expression is normalized against the housekeeping gene *rps11*. Changes in gene expression of TiLV-infected fish (black bars) are shown as *x*-fold increase compared with control fish at day 0 (white bars). The symbol (^*^) indicates significant differences between the control and TiLV-infected fish as revealed by the nonparametric Kruskal-Wallis test followed by Dunn’s test. The data are the mean + SD of *n* = 5–7 fish. ^*^ *p* ≤ 0.05; ^**^ *p* ≤ 0.01, ^***^ *p* ≤ 0.001, ^****^ *p* < 0.0001.

We observed that TiLV-infected zebrafish tend to group in the bottom of aquaria and almost do not react for feeding during progression of infection (**Supplementary Videos 1** and **2**). To assess the behavioral changes caused by TiLV infection, the following parameters were analyzed at 6 and 11 dpi: velocity (cm/s), distance traveled (cm), time in motion/motionless (s), and percentage of time spent in different zones (top *vs.* bottom) (%). Velocity test and distance moved test demonstrated a significant reduction in the speed and distance swam for TiLV-infected fish in comparison with mock-infected fish at 6 and 11 dpi (**Figures 3A, B**). Moreover, TiLV-infected fish tend to spend less time in-motion and more time in the bottom part of the tank when compared with mock-infected fish both at 6 and 11 dpi (**Figures 3A, B**). Furthermore, we observed that individual fish exhibited unusual spiral movement patterns during swimming.

The weight of infected fish systemically decreased as the infection progressed. A significant loss of body mass was demonstrated in infected animals at 18, 21, and 25 dpi (**Figure 3C**). Moreover, we observed a significant downregulation of *npy* (encoding orexigenic neuropeptide Y) at 14 and 28 dpi, with the strongest downregulation at 14 dpi (**Figure 3D**). We also noticed tendency to increase expression of *pomc* (encoding anorexigenic pro-opiomelanocortin) at 3 dpi, although this was not statistically significant (**Figure 3D**). These results show that TiLV infection leads to profound behavioral changes and may affect the appetite of infected fish.

### 3.4 Heads and Bodies of Zebrafish Larvae Showed Similar Viral Load and Antiviral Immune Response Against TiLV

**Figure 4:**
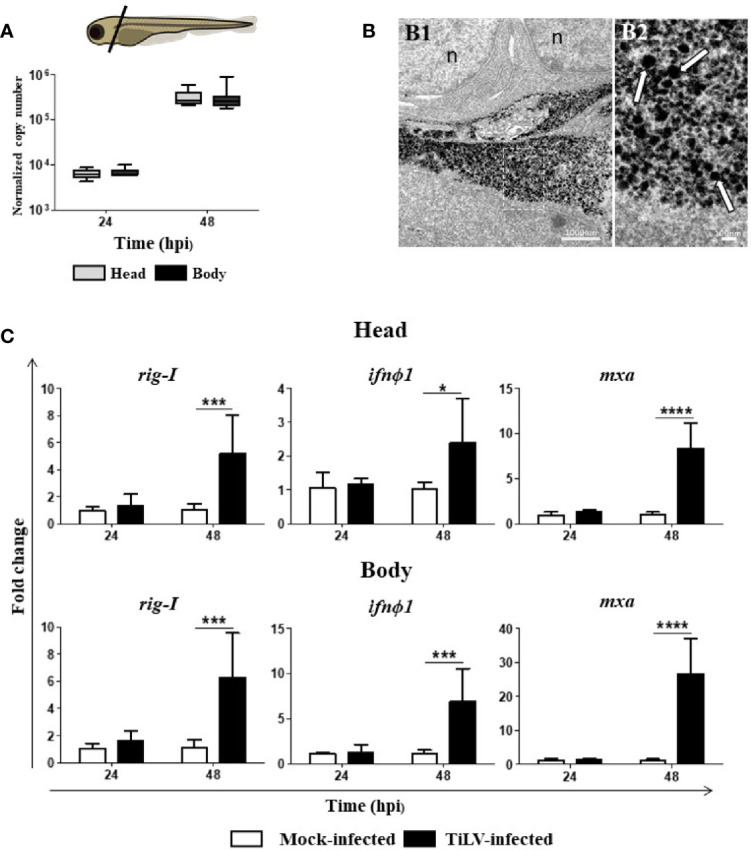
The presence of TiLV in the heads and bodies of zebrafish larvae and TiLV-induced changes in the expression of genes involved in antiviral response. **(A)** Normalized copy numbers of TiLV RNA in heads and bodies of TiLV-infected zebrafish larvae (*n* = 6–8). Zebrafish larvae were infected at the age of 54 hpf. The data are shown as box and whisker plots indicating the range of 25%–75% in the box and maximum and minimum values by whiskers. The line in the middle of the box is plotted at the median. The differences in viral load were analyzed using two-tailed nonparametric Mann-Whitney *U* test. Zebrafish larvae scheme was obtained from scidraw.io (doi.org/10.5281/zenodo.3925957). **(B)** Transmission electron micrographs of the brain from TiLV-infected zebrafish larvae showing cytoplasmic viral particles (white arrows) at low **(B1)** and high **(B2)** magnification. n, nucleus. **(C)** Expression of *rig-I*, *ifnϕ1*, and *mxa* in heads and bodies of zebrafish larvae. The gene expression is normalized against the housekeeping gene *rps11*. Changes in gene expression of TiLV-infected larvae (black bars) are shown as x-fold increase compared with mock-infected larvae (white bars) at each time point. The symbol (^*^) indicates significant differences between the mock-infected and TiLV-infected larvae at each time point as revealed by two-way ANOVA followed by a Bonferroni test (^*^ *p* ≤ 0.05; ^***^ *p* ≤ 0.001; ^****^ *p* < 0.0001). Each bar represents the mean + SD of *n* = 6–8 samples derived from two independent experiments.

We speculated that similar to TiLV-infected adult zebrafish, the larvae systemically infected by TiLV also exhibit higher viral load in the head region. However, quantification of TiLV by RT-qPCR showed no differences in viral load between the heads and bodies of zebrafish larvae at 24 and 48 hpi. Nevertheless, a high increase of the viral load at 48 hpi demonstrated that TiLV could probably replicate in both analyzed parts of zebrafish larvae (**Figure 4A**). The presence of TiLV virions in the brain of zebrafish larvae was also confirmed by TEM (**Figure 4B**). Finally, upregulation of the expression of *rig-I*, *ifnϕ1*, and *mxa* in both heads and bodies of zebrafish larvae were demonstrated during TiLV infection. The upregulation of studied genes was observed at 48 hpi and was higher in the bodies than in the heads (**Figure 4C**). These results demonstrated that at the first sampling points during TiLV infection of zebrafish larvae there are no differences in viral load between heads and bodies.

### 3.5 TiLV Causes Histopathological Abnormalities in the Brain of Zebrafish Larvae

**Figure 5:**
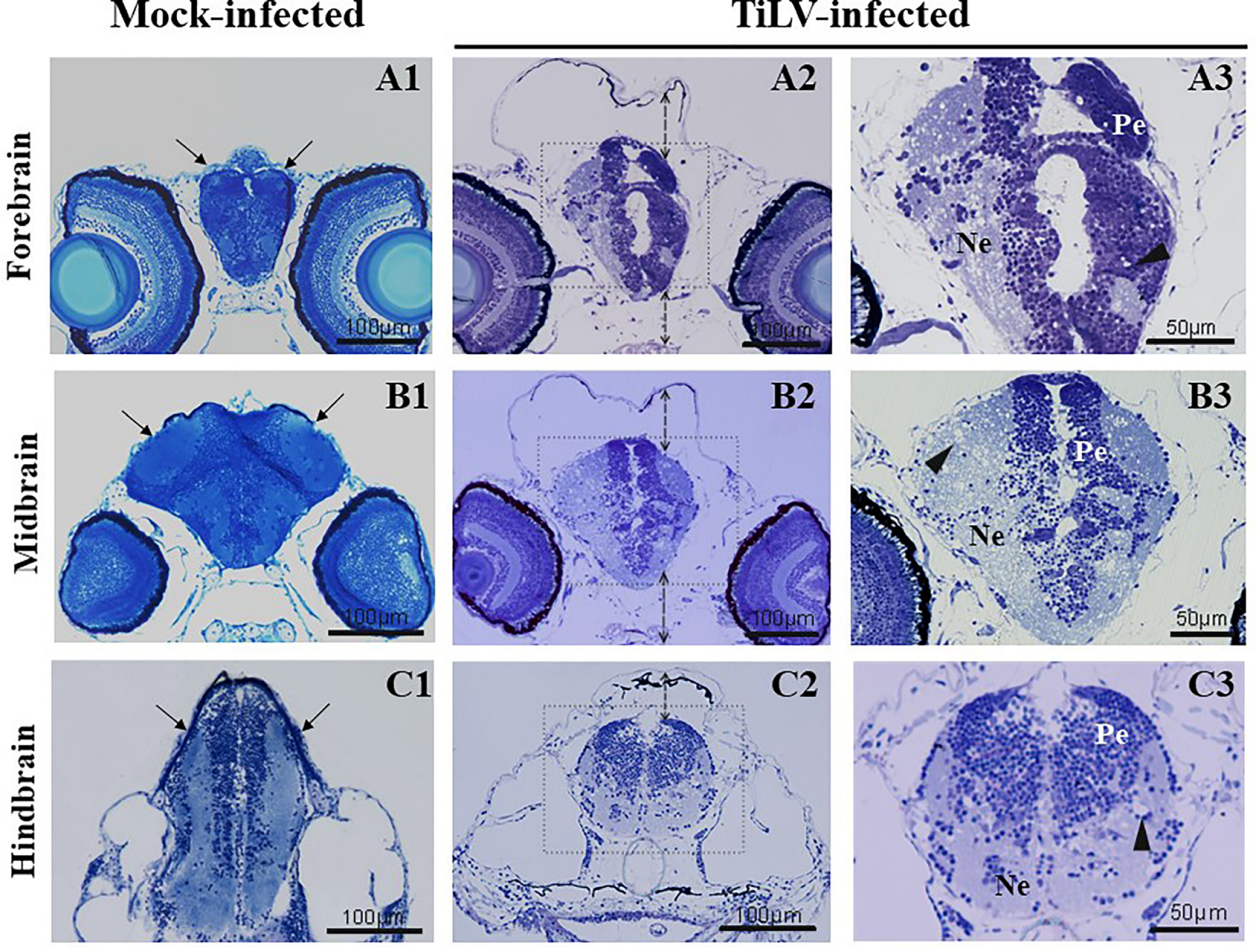
TiLV-induced histpathological changes in the brain of zebrafish larvae. The cross-sectioned head and brain sections of mock-infected **(A1**–**C1)** and TiLV-infected **(A2**–**C3)** zebrafish larvae at 48 hpi (102 hpf) stained with methylene blue/azur II. The solid lines with an arrow **(A1**–**C1)** show tight adhesion between brain and braincase. The dotted lines with an arrow **(A2**–**C2)** show enlarged free spaces (oedema) between the brain and braincase. **(A3**–**C3)** Higher magnification of the brain structure of TiLV-infected larvae with free alvealor spaces in the area of neuropil (arrowheads) and irregular distribution of nerve cell bodies. Ne, neuropil; Pe, perikarya.

The histological examination of the forebrain, midbrain and hindbrain area in mock-infected larvae revealed regular arrangement of the cell body of the neurons (or perikarya) and neuropil and tight adhesion of all tissues and organs, especially between brain, braincase, and sense organs (**Figures 5A1–C1**). The developing brain in TiLV-infected larvae in the area of the forebrain, midbrain, and hindbrain was surrounded by free space (or by fluid, oedema) causing the developing braincase is folded and protruding widely, especially at the dorsal side (**Figures 5A2–C2**). In all area of the brain in TiLV-infected larvae, the histopathological alterations concerning the degeneration in the neuropil and disintegration of the perikarya were visible (**Figures 5A3–C3**).

### 3.6 TiLV Induces Activation of Microglia in Zebrafish Larvae

**Figure 6:**
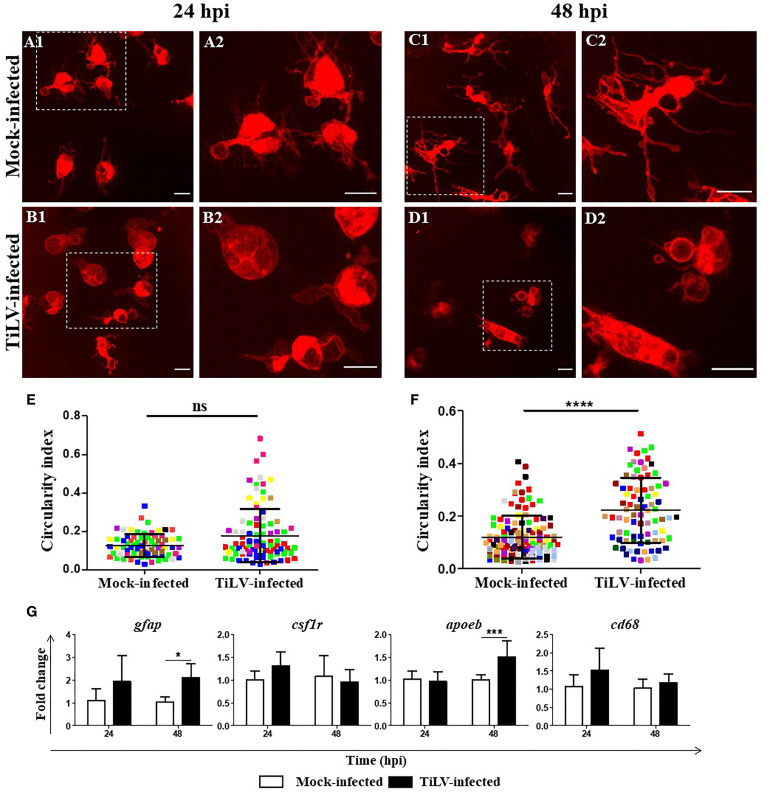
TiLV-induced microglia activation in zebrafish larvae. **(A–D)** Representative confocal images of microglia in the midbrain of transgenic *Tg(mpeg1.1:mCherryF)ump2* zebrafish larvae mock-infected at 24 hpi **(A1**, **A2)** and 48 hpi **(C1**, **C2)** or TiLV-infected at 24 hpi **(B1**, **B2)** and 48 hpi **(D1**, **D2)**. Zebrafish larvae were infected at the age of 54 hpf. Scale bar, 10 µm. The data are representative of two independent experiments. **(E, F)** Circularity index of microglia of zebrafish larvae at 24 hpi **(E)** (*n* = 8 larvae) and 48 hpi **(F)** (*n* = 15 larvae). Each dot represents a single microglia cell, and each color represents all microglia cells from single larvae. The symbol (^*^) indicates significant differences between the mock-infected and TiLV-infected zebrafish larvae as revealed by two-tailed nonparametric Mann-Whitney *U* test. The data derived from two independent experiments. **(G)** Expression of genes encoded astrocytes and microglia/macrophage markers in the heads of zebrafish larvae. The gene expression is normalized against the housekeeping gene *rps11*. Changes in gene expression of TiLV-infected larvae (black bars) are shown as *x*-fold increase compared with mock-infected larvae (white bars) at each time point. The symbol (^*^) indicates significant differences between the mock-infected and TiLV-infected larvae at each time point as revealed by two-way ANOVA followed by a Bonferroni test (^*^ *p* ≤ 0.05; ^***^ *p* ≤ 0.001; ^****^ *p* < 0.0001; ns, not significant). Each bar represents the mean + SD of *n* = 6–8 samples derived from two independent experiments.

Representative confocal images of microglia in the midbrain of TiLV-infected zebrafish larvae from transgenic *Tg(mpeg1.1:mCherryF)ump2* line showed that at 24 hpi, only a slight change in the morphology of microglia was present [**Figures 6A1, A2** (mock-infected) and **B1, B2** (TiLV-infected)]. However, at 48 hpi microglia clearly changed the shape from a resting ramified state in mock-infected (**Figures 6C1, C2**) to a highly ameboid active state in TiLV-infected larvae (**Figures 6D1, D2**). Quantification of the changes of microglia shape was performed by evaluation the circularity index. This analysis revealed no significant changes between mock-infected and TiLV-infected larvae at 24 hpi (**Figure 6E**), although the differences were statistically significant at 48 hpi (**Figure 6F**). Furthermore, gene expression studies revealed upregulation of the expression of the *gfap* and *apoeb* but not *csf1r* and *cd64*, at 48 hpi in the heads of zebrafish larvae (**Figure 6G**). These data demonstrated microglia activation during TiLV infection of zebrafish.

## 4 Discussion

Recent studies using molecular biology and genetic approaches demonstrated the role of viral infection in the development of neurobehavioral disorders and its broad impact on human and animal health and well-being. Moreover, these studies confirmed that the virus-induced changes in the nervous system might be induced by the virus itself or viral products; however, such changes may also be the result of the action of immune mediators produced in the brain during the antiviral response. In the present study, we prove that mechanisms involved in virus-induced immune response and sickness behavior are evolutionary conserved and fully operating already in the earliest vertebrates—fish. Using TiLV-zebrafish infection models (27, 28), we showed that TiLV exhibits neurotropism, remains at a high level in the brain for at least 3 months, and induces the type I IFN-dependent and inflammatory response in the CNS. Subsequently, in infected fish, we observed microglia activation and profound changes in the locomotor and feeding behavior.

Zebrafish is a laboratory model organism used in different areas of biological research, including studies of immune response and host-pathogen interactions. It has also been intensively used to study neurodevelopmental processes and neurodegenerative diseases (34), since its brain shows similarity to the mammalian brain both in general organization and cellular composition (35). However, little is known about zebrafish brain immune response during viral infection and the correlation between viral infection of the central nervous system and sickness behavior.

Previously, antiviral immune response in the brain was observed in fish (15, 16) and TiLV-induced changes in the brain were, to a limited extent, observed in infected Nile tilapia (26). In tilapia, it has been reported that during TiLV infection, viral titer in the brain is gradually increasing up to 17 dpi and remained high on 34 dpi (last studied time point). Moreover, ISH analysis revealed that TiLV was widely distributed in various parts of the tilapia brain, with the strongest signal observed in the forebrain (involved in learning and regulation of food intake) and the hindbrain (responsible for controlling locomotion and basal physiology) (36). In our study, the molecular analysis did not reveal any differences in the viral load in a specific part of the brain (forebrain, midbrain, and hindbrain) of adult zebrafish, while TEM analysis confirmed the presence of the virions in all three parts of the brain.

We have previously shown that zebrafish larvae appear to be more susceptible to TiLV as compared with adult fish, with high mortality observed already at 4–5 dpi (28). Using the zebrafish larvae infection model, we noticed viral replication in both the head and body of zebrafish larvae. However, we did not observe any differences in viral load between the head and body of the larvae at the studied time points (24 and 48 hpi). Although it is well known that young fish are more susceptible to virus infections as compared with adults (37), the future studies are required to determine whether TiLV also persist in the brain of surviving larvae and for how long it can be detected. Histopathological analysis of the brain showed clear pathological changes in the TiLV-infected larvae, including the degeneration in the neuropil and disintegration of the perikarya. Similarly, dissociation and degeneration within the infected sites of the brain of TiLV-infected tilapia has also been demonstrated (36). In adult zebrafish, we only observed enlarged blood vessels in the brain of TiLV-infected fish (14 dpi; data not shown). However, as we described previously (27), TiLV infection in adult zebrafish is less severe than that in tilapia and induces smaller histopathological changes in studied organs and very low mortality. Necrotic changes in the brain of adult zebrafish have been reported during infection with two neurotropic viruses: SVCV and NNV, which induce mortality of adult zebrafish (15, 16, 38). Moreover, NNV infection leads to extensive brain cell vacuolation in larvae of Asian seabass (*Lates calcarifer*) (39).

As mentioned before, the presence of TiLV in the brain of infected zebrafish induces neuroinflammation. In the brain of TiLV-infected zebrafish, we observed high upregulation of the expression of genes involved in type I IFN-dependent response and genes encoding proinflammatory mediators. In line with our results, upregulation of the expression of *tlr3*, *ifn-β*, and *mx* genes in the brain of TiLV-infected Nile tilapia has been demonstrated (26). Interestingly, TiLV downplayed innate immune response during early stage of infection in tilapia (26), which was not observed in our study. Such differences could be partly explained by the different animal species used for TiLV study. Previous reports have revealed the activation of type I IFN-dependent response and induction of inflammation in zebrafish brain upon infection with NNV and SVCV (15, 16) and during nodavirus infections of sea bream (*Sparus aurata*) and sea bass (*Dicentrarchus labrax*) (40).

Both “brain-borne” and circulating proinflammatory cytokines may act at the level of CNS and induce sickness behavior (4, 5, 41). Previous studies have demonstrated that TiLV-infected tilapia displays a loss of appetite, lethargy, erratic swimming, loss of balance, swimming at the water surface, stoppage of schooling, or swirling (24, 29, 36, 42). Here, we demonstrated that zebrafish infected with TiLV had reduced velocity, shortened travelled distance, reduced time in-motion, and increased time spent at the bottom zone of aquaria. This indicates that TiLV induces a clear sickness behavior of zebrafish. Previously, sickness behavior in zebrafish has also been observed during an inflammatory response induced by the inoculation of formalin-inactivated *Aeromonas hydrophila* (43). Fish with an induced immune response showed reduced locomotor activity, changes in their social preference, and exploratory behavior toward a new object compared with control fish (43). Furthermore, we found a correlation between progression of TiLV infection and weight loss of zebrafish. As in mammals, food intake in fish is under the control of the feeding system, located in the brain, and is regulated by several specific protein molecules including neuropeptides, gastrointestinal peptides, hormones, and blood metabolites (44). Reduced food intake is a common feature of sick fish and cytokines appear to be involved in this process (45). After examining a broad panel of genes involved in the control of food intake in fish, we found TiLV-induced downregulation of *npy*, which encodes orexigenic neuropeptide Y.

Research in mammals suggests that, although peripherally produced cytokines may cross the blood-brain barrier, the main source of these molecules in the brain are activated glial cells (microglia and astrocytes) (46). In addition, these cells are involved in the neuroinflammatory processes that can lead to brain pathologies (47, 48). On the other hand, a mouse model of acute viral encephalitis suggests the protective role of microglia in the virus-driven pathogenic neuroinflammation and indicates the role of these cells in the limiting of viral replication (11). Still, little is known regarding the microglial activation during viral infection in fish. In zebrafish, microglia activation was mainly studied in the context of developmental and regeneration processes including phagocytosis of dying neurons (49). Moreover, an interesting response of microglia during bacterial infection with *Mycobacterium marinum* was reported in zebrafish larvae (50). The authors suggested that microglia were able to reverse migrate to join infection foci in the body and to combat the *M. marinum* infection together with peripheral macrophages. Conversely, the early larval hindbrain injection with bacteria mobilized peripheral macrophages into the infection site (50). Chiang and coworkers demonstrated that NNV infection of the primary culture of brain cells from giant grouper (*Epinephelus lanceolatus*) activates microglial cell proliferation and secretion of proinflammatory cytokines (IL-1β and TNF-α) and subsequently the neural cell death (51). Involvement of microglia in neuroinflammation in fish upon bacterial infection with *Weissella cibaria* has also been described *in vitro* using microglia cultures isolated from the brain of Nile tilapia (52).

To understand the dynamics of the processes of microglia activation during infection/inflammation, it is necessary to observe them in a broader context of CNS in the living organism. Zebrafish is an excellent model for such studies as microglia can be visualised *in vivo* using transgenic zebrafish-expressing fluorescent proteins driven by promoters of microglia/macrophage-specific genes such as *mpeg1* (53), *apoe* (54), or microglia-specific purinergic receptor *p2y12* (55). These studies have been mainly performed using zebrafish larvae model due to its transparency, which allows to observe microglia in their natural environment under very rapid changes. In contrast, most of the experimental approach using adult zebrafish include brain fixation. The experimental data show that methods based on *ex vivo* analysis of brain samples after different ways of fixation can greatly affect the microglial morphology due to even small ischemic conditions upon preparations (56). Therefore, in this case, methodology can artificially affect the conclusions of the immune status of these highly dynamic cells. Methods like live, real-time imaging on adult zebrafish brain has been described (57); however, it is rarely used due to technical challenges such as demand for a custom-built point-scanning upright microscope (57). Therefore, in the present studies, we decided to use well-described protocol (53) of *in vivo* microglia observation in transgenic *Tg(mpeg1.1:mCherryF)ump2* zebrafish larvae. It has to be mentioned that primitive macrophages from the yolk sac colonize the zebrafish brain from 48 hpf and rapidly differentiate over the next 24 h (58). In the process of maturation, larval microglia significantly upregulate the expression of microglial core genes such as *apoeb*, *p2ry12*, *hexb*, and *csf1r* (59) and downregulate the expression of *lcp1* (*L-plastin*) (60). Even though the core microglia genes and their expression during microglia maturation have been studied in fish, little is known regarding the markers that indicate their activation during infection. Here, we demonstrated that TiLV infection induces an upregulation of the expression of microglia/macrophage markers *csf1r* and *cd68* in the brain of adult fish and *apoeb* in the larvae as well as the astrocyte marker *gfap* in the larvae. In mammals, the activation of microglia during neuronal death, injury, or infection, leads to the changes of the expression of genes encoding specific cell markers but also to the change of their morphology. Activated microglia have a circular, highly motile, and phagocytic phenotype (61). In fish, microglia activation has been investigated mainly during brain injury (62). Here, we demonstrated for the first time the activation of microglia during viral infection. TiLV-activated microglia changed their shape from highly ramified cells present in control larvae, to ameboid, spherical morphology. Similar phenotype of activated microglia has been reported in zebrafish due to phagocytosis of neurons (54) or bacteria (63). It remains to be determined whether the activated microglia observed in our study are also involved in the process of phagocytosis brain cells possibly damaged by TiLV.

In conclusion, this is the first study presenting a comprehensive analysis of the brain immune response connected with microglia activation and subsequent sickness behavior during systemic viral infection of zebrafish. We believe that the extended knowledge of evolutionary conservation of important ligands and receptors involved in the regulation of the immune response within the nervous system and in inducing behavioral changes will support the hypothesis regarding the functional importance of these. In this context, zebrafish forms a perfect model to identify fundamental ligands and receptors and their evolutionary relationships.

## Data Availability Statement

The original contributions presented in the study are included in the article/**Supplementary Material**. Further inquiries can be directed to the corresponding author.

## Ethics Statement

The animal study was reviewed and approved by the 2nd Local Ethics Committee in Kraków, Poland.

## Author Contributions

KR and MC conceived the study. KR acquired the funding. MM and MW performed the infection and behavioral experiments with help from NP. MM and UO performed gene expression study. MA performed viral load analysis. PP analyzed results from behavioral experiments. TP and MW performed confocal microscopy. MW analyzed circularity index. AP performed histopathology study. AM performed TEM analysis. WS provided TiLV. MM performed statistical analysis. MM, MW, MC, and KR wrote the main body of the manuscript with contributions from all other authors. All authors contributed to the article and approved the submitted version.

## Funding

This work was supported by the National Science Centre of Poland within Sonata Bis 5 project (Grant number UMO-2015/18/E/NZ6/00516). TP was supported by the NCN Sonata Bis 9 project (Grant number UMO-2019/34/E/NZ6/00137). The open-access publication of this article was funded by the Priority Research Area BioS under the program “Excellence Initiative – Research University” at the Jagiellonian University in Krakow.

## Conflict of Interest

The authors declare that the research was conducted in the absence of any commercial or financial relationships that could be construed as a potential conflict of interest.

## Publisher’s Note

All claims expressed in this article are solely those of the authors and do not necessarily represent those of their affiliated organizations, or those of the publisher, the editors and the reviewers. Any product that may be evaluated in this article, or claim that may be made by its manufacturer, is not guaranteed or endorsed by the publisher.

## Supplementary Material

Supplementary Videos 1 and 2 Adult zebrafish were mock-infected (movie 1) or TiLV-infected (movie 2). Fish were fed twice per day with commercial feed for zebrafish (Gemma Micro 300ZF, Skretting). Observation of the feeding behavior demonstrated that starting from 6 dpi TiLV-infected fish almost do not react for feeding and tend to group in the bottom part of the aquarium. Movies were recorded at 11 dpi.

## References

[1] Dantzer R Kelley KW. Twenty Years of Research on Cytokine-Induced Sickness Behavior. Brain Behav Immun (2007) 21(2):153–60. doi: 10.1016/j.bbi.2006.09.006 PMC1850954 17088043

[2] Grossberg AJ Zhu X Leinninger GM Levasseur PR Braun TP Myers MG. Inflammation-Induced Lethargy is Mediated by Suppression of Orexin Neuron Activity. J Neurosci (2011) 31(31):11376–86. doi: 10.1523/JNEUROSCI.2311-11.2011 PMC3155688 21813697

[3] Haba R Shintani N Onaka Y Wang H Takenaga R Hayata A. Lipopolysaccharide Affects Exploratory Behaviors Toward Novel Objects by Impairing Cognition and/or Motivation in Mice: Possible Role of Activation of the Central Amygdala. Behav Brain Res (2012) 228(2):423–31. doi: 10.1016/j.bbr.2011.12.027 22209851

[4] Kelley KW Bluthé RM Dantzer R Zhou JH Shen WH Johnson RW. Cytokine-Induced Sickness Behavior. Brain Behav Immun (2003) 1:S112–118. doi: 10.1016/S0889-1591(02)00077-6 12615196

[5] Dantzer R. Cytokine, Sickness Behavior, and Depression. Neurol Clin (2006) 24(3):441–60. doi: 10.1016/j.ncl.2006.03.003 PMC2909644 16877117

[6] Dantzer R O’Connor JC Freund GG Johnson RW Kelley KW. From Inflammation to Sickness and Depression: When the Immune System Subjugates the Brain. Nat Rev Neurosci (2008) 9(1):46–56. doi: 10.1038/nrn2297 18073775 PMC2919277

[7] Das T Hoarau JJ Bandjee MCJ Maquart M Gasque P. Multifaceted Innate Immune Responses Engaged by Astrocytes, Microglia and Resident Dendritic Cells Against Chikungunya Neuroinfection. J Gen Virol (2015) 96(Pt2):294–310. doi: 10.1099/vir.0.071175-0 25351727

[8] Paolicelli RC Gross CT. Microglia in Development: Linking Brain Wiring to Brain Environment. Neuron Glia Biol (2011) 7(1):77–83. doi: 10.1017/S1740925X12000105 22857738

[9] Schlegelmilch T Henke K Peri F. Microglia in the Developing Brain: From Immunity to Behaviour. Curr Opin Neurobiol (2011) 21(1):5–10. doi: 10.1016/j.conb.2010.08.004 20817438

[10] Rock RB Gekker G Hu S Sheng WS Cheeran M Lokensgard JR. Role of Microglia in Central Nervous System Infections. Clin Microbiol Rev (2004) 17(4):942–64. doi: 10.1128/CMR.17.4.942-964.2004 PMC523558 15489356

[11] Hatton CF Duncan CJA. Microglia are Essential to Protective Antiviral Immunity: Lessons From Mouse Models of Viral Encephalitis. Front Immunol (2019) 10:2656. doi: 10.3389/fimmu.2019.02656 31798586 PMC6863772

[12] Levraud JP Palha N Langevin C Boudinot P. Through the Looking Glass: Witnessing Host-Virus Interplay in Zebrafish. Trends Microbiol (2014) 22(9):490–7. doi: 10.1016/j.tim.2014.04.014 24865811

[13] Rakus K Adamek M Mojżesz M Podlasz P Chmielewska-Krzesińska M Naumowicz K. Evaluation of Zebrafish (*Danio Rerio*) as an Animal Model for the Viral Infections of Fish. J Fish Dis (2019) 42(6):923–34. doi: 10.1111/jfd.12994 30920010

[14] Varela M Figueras A Novoa B. Modelling Viral Infections Using Zebrafish: Innate Immune Response and Antiviral Research. Antivir Res (2017) 139:59–68. doi: 10.1016/j.antiviral.2016.12.013 28025085

[15] Lu MW Chao YM Guo TC Santi N Evensen Ø Kasani SK. The Interferon Response is Involved in Nervous Necrosis Virus Acute and Persistent Infection in Zebrafish Infection Model. Mol Immunol (2008) 45(4):1146–52. doi: 10.1016/j.molimm.2007.07.018 17727953

[16] Wang Y Zhang H Lu Y Wang F Liu L Liu J. Comparative Transcriptome Analysis of Zebrafish (*Danio Rerio*) Brain and Spleen Infected With Spring Viremia of Carp Virus (SVCV). Fish Shellfish Immunol (2017) 69:35–45. doi: 10.1016/j.fsi.2017.07.055 28757199

[17] Boltaña S Rey S Roher N Vargas R Huerta M Huntingford FA. Behavioural Fever is a Synergic Signal Amplifying the Innate Immune Response. Proc R Soc B (2013) 280(1766):20131381. doi: 10.1098/rspb.2013.1381 PMC3730603 23843398

[18] Rey S Moiche V Bolta S Teles M Mackenzie S. Behavioural Fever in Zebrafish Larvae. Dev Comp Immunol (2017) 67:287–92. doi: 10.1016/j.dci.2016.09.008 27670815

[19] Burgos JS Ripoll-Gomez J Alfaro JM Sastre I Valdivieso F. Zebrafish as a New Model for Herpes Simplex Virus Type 1 Infection. Zebrafish (2008) 5(4):323–33. doi: 10.1089/zeb.2008.0552 19133831

[20] Palha N Guivel-Benhassine F Briolat V Lutfalla G Sourisseau M Ellett F. Real-Time Whole-Body Visualization of Chikungunya Virus Infection and Host Interferon Response in Zebrafish. PloS Pathog (2013) 9(9):e1003619. doi: 10.1371/journal.ppat.1003619 24039582 PMC3764224

[21] Passoni G Langevin C Palha N Mounce BC Briolat V Affaticati P. Imaging of Viral Neuroinvasion in the Zebrafish Reveals That Sindbis and Chikungunya Viruses Favour Different Entry Routes. Dis Model Mech (2017) 10(7):847–57. doi: 10.1242/dmm.029231 PMC5536907 28483796

[22] Bacharach E Mishra N Briese T Zody MC Tsofack JEK Zamostiano R. Characterization of a Novel Orthomyxo-Like Virus Causing Mass Die-Offs of Tilapia. mBio (2016) 7(2):e00431–16. doi: 10.1128/mBio.00431-16 PMC4959514 27048802

[23] Eyngor M Zamostiano R Kembou Tsofack JEK Berkowitz A Bercovier H Tinman S. Identification of a Novel RNA Virus Lethal to Tilapia. J Clin Microbiol (2014) 52(12):4137–46. doi: 10.1128/JCM.00827-14 PMC4313277 25232154

[24] Surachetpong W Roy SRK Nicholson P. Tilapia Lake Virus: The Story So Far. J Fish Dis (2020) 43(10):1115–32. doi: 10.1111/jfd.13237 32829488

[25] Mugimba KK Tall S Dubey S Mutoloki S Dishon A Evensen Q. Gray *(Oreochromis Niloticus* X O. Aureus) and Red (*Oreochromis Spp.*) Tilapia Show Equal Susceptibility and Proinflammatory Cytokine Responses to Experimental. Viruses (2019) 11(10):893. doi: 10.3390/v11100893 PMC6832934 31554184

[26] Mugimba KK Lamkhannat M Dubey S Mutoloki S Munang’andu HM Evensen Ø. Tilapia Lake Virus Downplays Innate Immune Responses During Early Stage of Infection in Nile Tilapia (*Oreochromis Niloticus*). Sci Rep (2020) 10(1):20364. doi: 10.1038/s41598-020-73781-y 33230226 PMC7684318

[27] Rakus K Mojzesz M Widziolek M Pooranachandran N Teitge F Surachetpong W. Antiviral Response of Adult Zebrafish (*Danio Rerio*) During Tilapia Lake Virus (TiLV) Infection. Fish Shellfish Immunol (2020) 101:1–8. doi: 10.1016/j.fsi.2020.03.040 32201348

[28] Widziolek M Janik K Mojzesz M Pooranachandran N Adamek M Pecio A. Type I Interferon-Dependent Response of Zebrafish Larvae During Tilapia Lake Virus (TiLV) Infection. Dev Comp Immunol (2021) 116:103936. doi: 10.1016/j.dci.2020.103936 33242567

[29] Tattiyapong P Dachavichitlead W Surachetpong W. Experimental Infection of Tilapia Lake Virus (TiLV) in Nile Tilapia (*Oreochromis Niloticus*) and Red Tilapia (Oreochromis Spp. ) Vet Microbiol (2017) 207:170–7. doi: 10.1016/j.vetmic.2017.06.014 28757020

[30] Bernut A Herrmann JL Kissa K Dubremetz JF Gaillard JL Lutfalla G. *Mycobacterium Abscessus* Cording Prevents Phagocytosis and Promotes Abscess Formation. Proc Natl Acad Sci U.S.A. (2014) 11(10):E943–52. doi: 10.1073/pnas.1321390111 PMC3956181 24567393

[31] Feng H Zhang QM Zhang YB Li Z Zhang J Xiong YW. Zebrafish IRF1, IRF3, and IRF7 Differentially Regulate IFNΦ1 and IFNΦ3 Expression Through Assembly of Homo- or Heteroprotein Complexes. J Immunol (2016) 197(5):1893–904. doi: 10.4049/jimmunol.1600159 27496972

[32] Pfaffl MW. A New Mathematical Model for Relative Quantification in Real-Time RT-PCR. Nucleic Acids Res (2001) 29(9):e45. doi: 10.1093/nar/29.9 11328886 PMC55695

[33] Young K Morrison H. Quantifying Microglia Morphology From Photomicrographs of Immunohistochemistry Prepared Tissue Using ImageJ. J Vis Exp (2018) 136:57648. doi: 10.3791/57648 PMC6103256 29939190

[34] Stewart AM Braubach O Spitsbergen J Gerlai R Kalueff AV. Zebrafish Models for Translational Neuroscience Research: From Tank to Bedside. Trends Neurosci (2014) 37(5):264–78. doi: 10.1016/j.tins.2014.02.011 PMC4039217 24726051

[35] Kalueff AV Stewart AM Gerlai R. Zebrafish as an Emerging Model for Studying Complex Brain Disorders. Trends Pharmacol Sci (2014) 35(2):63–75. doi: 10.1016/j.tips.2013.12.002 24412421 PMC3913794

[36] Dinh-Hung N Sangpo P Kruangkum T Kayansamruaj P Rung-Ruangkijkrai T Senapin S. Dissecting the Localization of Tilapia Tilapinevirus in the Brain of the Experimentally Infected Nile Tilapia, *Oreochromis Niloticus* (L.). J Fish Dis (2021) 44(8):1053–64. doi: 10.1111/jfd.13367 33724491

[37] Bergmann SM Fichtner D Skall HF Schlotfeldt HJ Olesen NJ. Age-And Weight-Dependent Susceptibility of Rainbow Trout *Oncorhynchus Mykiss* to Isolates of Infectious Haematopoietic Necrosis Virus (IHNV) of Varying Virulence. Dis Aquat Organ (2003) 55(3):205–10. doi: 10.3354/dao055205 13677506

[38] Binesh CP. Mortality Due to Viral Nervous Necrosis in Zebrafish *Danio Rerio* and Goldfish Carassius Auratus. Dis Aquat Organ (2013) 104(3):257–60. doi: 10.3354/dao02605 23759563

[39] Ransangan J Manin BO. Mass Mortality of Hatchery-Produced Larvae of Asian Seabass, *Lates Calcarifer* (Bloch), Associated With Viral Nervous Necrosis in Sabah, Malaysia. Vet Microbiol (2010) 145(1-2):153–7. doi: 10.1016/j.vetmic.2010.03.016 20427132

[40] Poisa-Beiro L Dios S Montes A Aranguren R Figueras A Novoa B. Nodavirus Increases the Expression of Mx and Inflammatory Cytokines in Fish Brain. Mol Immunol (2008) 45(1):218–25. doi: 10.1016/j.molimm.2007.04.016 17543386

[41] Tizard I. Sickness Behavior, its Mechanisms and Significance. Anim Health Res Rev (2008) 9(1):87–99. doi: 10.1017/S1466252308001448 18423072

[42] Jansen MD Dong HT Mohan CV. Tilapia Lake Virus: A Threat to the Global Tilapia Industry? Rev Aquac (2019) 11:725–39. doi: 10.1111/raq.12254

[43] Kirsten K Soares SM Koakoski G Carlos Kreutz L Barcellos LJG. Characterization of Sickness Behavior in Zebrafish. Brain Behav Immun (2018) 73:596–602. doi: 10.1016/j.bbi.2018.07.004 29981831

[44] Volkoff H. The Neuroendocrine Regulation of Food Intake in Fish: A Review of Current Knowledge. Front Neurosci (2016) 10:540. doi: 10.3389/fnins.2016.00540 27965528 PMC5126056

[45] Volkoff H Peter RE. Effects of Lipopolysaccharide Treatment on Feeding of Goldfish: Role of Appetite-Regulating Peptides. Brain Res (2004) 998(2):139–47. doi: 10.1016/j.brainres.2003.11.011 14751584

[46] Pinteaux E Parker LC Rothwell NJ Luheshi GN. Expression of Interleukin-1 Receptors and Their Role in Interleukin-1 Actions in Murine Microglial Cells. J Neurochem (2002) 83(4):754–63. doi: 10.1046/j.1471-4159.2002.01184.x 12421347

[47] Carmignoto G. Reciprocal Communication Systems Between Astrocytes and Neurones. Prog Neurobiol (2000) 62(6):561–81. doi: 10.1016/s0301-0082(00)00029-0 10880851

[48] Mandrekar-Colucci S Landreth GE. Microglia and Inflammation in Alzheimer's Disease. CNS Neurol Disord Drug Targets (2010) 9(2):156–67. doi: 10.2174/187152710791012071 PMC3653290 20205644

[49] Var SR Byrd-Jacobs CA. Role of Macrophages and Microglia in Zebrafish Regeneration. Int J Mol Sci (2020) 21(13):4768. doi: 10.3390/ijms21134768 PMC7369716 32635596

[50] Davis JM Clay H Lewis JL Ghori N Herbomel P Ramakrishnan L. Real-Time Visualization of *Mycobacterium-*Macrophage Interactions Leading to Initiation of Granuloma Formation in Zebrafish Embryos. Immunity (2002) 17(6):693–702. doi: 10.1016/s1074-7613(02)00475-2 12479816

[51] Chiang YH Wu YC Chi SC. Interleukin-1β Secreted From Betanodavirus-Infected Microglia Caused the Death of Neurons in Giant Grouper Brains. Dev Comp Immunol (2017) 70:19–26. doi: 10.1016/j.dci.2017.01.002 28062227

[52] Eto SF Fernandes DC Funnicelli MIG Alecrim JVC Souza PG Carvalho FCA. Microglia Extracellular Traps in *Oreochromis Niloticus* Infected With Weissella Cibaria. Fish Shellfish Immunol (2021) 113:148–53. doi: 10.1016/j.fsi.2021.03.020 33838222

[53] Hamilton N Rutherford HA Petts JJ Isles HM Weber T Henneke M. The Failure of Microglia to Digest Developmental Apoptotic Cells Contributes to the Pathology of RNASET2-Deficient Leukoencephalopathy. Glia (2020) 68(7):1531–45. doi: 10.1002/glia.23829 PMC8647916 32212285

[54] Peri F Nüsslein-Volhard C. Live Imaging of Neuronal Degradation by Microglia Reveals a Role for V0-ATPase A1 in Phagosomal Fusion *In Vivo* . Cell (2008) 133(5):916–27. doi: 10.1016/j.cell.2008.04.037 18510934

[55] Herzog C Pons Garcia L Keatinge M Greenald D Moritz C Peri F. Rapid Clearance of Cellular Debris by Microglia Limits Secondary Neuronal Cell Death After Brain Injury *In Vivo* . Development (2019) 146(9):dev174698. doi: 10.1242/dev.174698 31076485 PMC6526721

[56] Cătălin B Stopper L Bălşeanu T-A Scheller A. The *in Situ* Morphology of Microglia is Highly Sensitive to the Mode of Tissue Fixation. J Chem Neuroanat (2017) 86:59–66. doi: 10.1016/j.jchemneu.2017.08.007 28866082

[57] Dray N Bedu S Vuillemin N Alunni A Coolen M Krecsmarik M. Large-Scale Live Imaging of Adult Neural Stem Cells in Their Endogenous Niche. Development (2015) 142(20):3592–600. doi: 10.1242/dev.123018 PMC4631764 26395477

[58] Xu J Wang T Wu Y Jin W Wen Z. Microglia Colonization of Developing Zebrafish Midbrain is Promoted by Apoptotic Neuron and Lysophosphatidylcholine. Dev Cell (2016) 38(2):214–22. doi: 10.1016/j.devcel.2016.06.018 27424497

[59] Mazzolini J Le Clerc S Morisse G Coulonges C Kuil LE van Ham TJ. Gene Expression Profiling Reveals a Conserved Microglia Signature in Larval Zebrafish. Glia (2020) 68(2):298–315. doi: 10.1002/glia.23717 31508850 PMC6916425

[60] Herbomel P Thisse B Thisse C. Zebrafish Early Macrophages Colonize Cephalic Mesenchyme and Developing Brain, Retina, and Epidermis Through a M-CSF Receptor-Dependent Invasive Process. Dev Biol (2001) 238(2):274–88. doi: 10.1006/dbio.2001.0393 11784010

[61] Pinto MV Fernandes A. Microglial Phagocytosis-Rational But Challenging Therapeutic Target in Multiple Sclerosis. Int J Mol Sci (2020) 21(17):5960. doi: 10.3390/ijms21175960 PMC7504120 32825077

[62] Gan D Wu S Chen B Zhang J. Application of the Zebrafish Traumatic Brain Injury Model in Assessing Cerebral Inflammation. Zebrafish (2020) 17(2):73–82. doi: 10.1089/zeb.2019.1793 31825288

[63] Wu S Nguyen LTM Pan H Hassan S Dai Y Xu J. Two Phenotypically and Functionally Distinct Microglial Populations in Adult Zebrafish. Sci Adv (2020) 6(47):eabd1160. doi: 10.1126/sciadv.abd1160 33208372 PMC7673811

